# Separation of Allelopathy from Resource Competition Using Rice/Barnyardgrass Mixed-Cultures

**DOI:** 10.1371/journal.pone.0037201

**Published:** 2012-05-10

**Authors:** Hai Bin He, Hai Bin Wang, Chang Xun Fang, Zhi Hua Lin, Zheng Ming Yu, Wen Xiong Lin

**Affiliations:** 1 Key Laboratory of Biopesticide and Chemical Biology, Ministry of Education, and Agroecological Institute/School of Life Sciences, Fujian Agriculture and Forestry University, Fuzhou, China; 2 Agroecological Institute/School of Life Sciences, Fujian Agriculture and Forestry University, Fuzhou, China; Centro de Investigación y de Estudios Avanzados del IPN, Mexico

## Abstract

Plant-plant interference is the combined effect of allelopathy, resource competition, and many other factors. Separating allelopathy from resource competition is almost impossible in natural systems but it is important to evaluate the relative contribution of each of the two mechanisms on plant interference. Research on allelopathy in natural and cultivated plant communities has been hindered in the absence of a reliable method that can separate allelopathic effect from resource competition. In this paper, the interactions between allelopathic rice accession PI312777, non-allelopathic rice accession Lemont and barnyardgrass were explored respectively by using a target (rice)-neighbor (barnyardgrass) mixed-culture in hydroponic system. The relative competitive intensity (RCI), the relative neighbor effect (RNE) and the competitive ratio (CR) were used to quantify the intensity of competition between each of the two different potentially allelopathic rice accessions and barnyardgrass. Use of hydroponic culture system enabled us to exclude any uncontrolled factors that might operate in the soil and we were able to separate allelopathy from resource competition between each rice accession and barnyardgrass. The RCI and RNE values showed that the plant-plant interaction was positive (facilitation) for PI312777 but that was negative (competition) for Lemont and barnyardgrass in rice/barnyardgrass mixed-cultures. The CR values showed that one PI312777 plant was more competitive than 2 barnyardgrass plants. The allelopathic effects of PI312777 were much more intense than the resource competition in rice/barnyardgrass mixed cultures. The reverse was true for Lemont. These results demonstrate that the allelopathic effect of PI312777 was predominant in rice/barnyardgrass mixed-cultures. The most significant result of our study is the discovery of an experimental design, target-neighbor mixed-culture in combination with competition indices, can successfully separate allelopathic effects from competition.

## Introduction

Plants can affect neighboring plants by releasing chemicals into the environment. The Austrian plant physiologist Hans Molish named this phenomenon, “allelopathy" in 1937. The existence of allelopathy has been well documented over the past few decades in both natural and agricultural ecosystems [1], [2], [3]. However, the study of allelopathy has provoked so much controversy that some authors still question its existence. This is mainly because traditionally plant-plant interactions have been considered to be predominantly mediated by competition for limited resources. According to Mallik, the mainstream ecologists practically ignored research on allelopathy, based on the argument that in most allelopathy research the influence of other major factors such as resource competition, soil chemical and biological properties are not considered and successfully eliminated to demonstrate the effect of allelopathy [2]. This is partly due to a lack of reliable techniques that can separate allelopathic influences from other forms of plant interference, and partly due to the complex nature of allelopathic effects under natural conditions. Muller addressed this problem by summing up the effects of allelopathy and competition and proposed plant interference model [4]. Putnam and Duke later suggested that allelopathy can be separated from other mechanisms of plant interference in that any detrimental effect is exerted through the release of a chemical by the donor [5]. Reigosa et al. are of the opinion that the ecophysiological point of view must be considered if we are to obtain defendable results and valid conclusions about the role of allelopathy in nature [6].

In rice (*Oriza sativ*a L.) cultivation, the presence of weeds is a persistent problem. Even at a ratio of 100 rice plants to 10 barnyardgrass (BYG) plants, rice biomass is reduced by 75% and yield is lessened by about 50% [7]. BYG has been proven to be a better competitor when both rice and BYG are transplanted at roughly similar phenological stages. This is mainly for its faster development and greater height [7]. Synthetic herbicides are the only tool available for BYG control. Due to the negative effects of synthetic herbicides, such as herbicide-resistant weeds, environmental contamination, and human health problems, there have been considerable efforts in designing alternative weed management strategies. Allelopathy is considered a good weed management tool for the production of weed-resistant crops. Putnam and Duke suggested utilizing allelopathic crops to suppress weed growth in agricultural systems [8]. Dilday et al. discovered a weed-free zone surrounding an allelopathic rice cultivar, Taichung Native 1 [9], [10]. This plant showed an allelopathic effect against four weed species. Rice researchers have turned their interests in rice allelopathy in the hope of combating weeds and reducing/eliminating synthetic herbicide use in rice production, thereby decreasing their negative effects on agroecosystems. Allelopathy is not an isolated phenomenon in natural ecosystems. It works with resource competition and many other ecophysiological processes interacting simultaneously. The difficulty of distinguishing chemical interference from competition has hindered studies of allelopathy in natural and cultivated plant communities [11]. Inderjit and del Moral suggested that separating allelopathy from resource competition is almost impossible in natural systems but the relative contribution of the two mechanisms on plant interference is possible to determine and important to do so [12]. Allelopathic rice cause weed inhibition at its early developmental stage [13], [14]. Weeding in the first 30 days following transplanting is important [15]. Better understanding on the nature of interactions between allelopathic rice and weeds might enhance the ability of rice seedlings to compete and reduce the use of synthetic herbicides [16].

Some researchers have made useful contributions to distinguishing allelopathy and other mechanisms involved in plant-plant interactions. Weidenhamer et al. quantified the biomass of bahiagrass (*Paspalum notatum* Fluega) grown in soil treated with hydroquine and gallic acid and that of tomatoes (*Lycopersicon esculentum* Mill.) grown in soils taken from under and around black walnut trees (*Juglans nigra* L.) [17]. Their results suggested that analysis of a density-dependent approach can help distinguish resource competition and allelopathy. Used target-neighbor design and atrazine as a phytotoxin, Thijs et al. studied the competitive outcome of corn-soybean mixtures [18]. Their results showed this to be an effective experimental design for allelopathy study. Using PVC pipes to reduce root competition and activated carbon to reduce allelopathy, Nilsson showed that allelopathy and competition of *Empetrum hermaphroditum* can be separated and quantified [19]. Weidenhamer suggested that distinguishing allelopathy from other forms of plant-plant interactions is a better approach than attempting to separate them [20]. Not many studies reported on the relationship between allelopathy and resource competition with respect to allelopathic rice accessions and weeds. Olofsdotter opined that distinguish allelopathy from competition is necessary to optimize both effects and maximize weed reduction [13].

Rice [21] defined allelopathy as any direct or indirect effect by one plant (including microorganisms) on another through production of chemical compounds that escape into the environment. This definition has been modified by the International Allelopathy Society to – any process involving secondary metabolites produced by plants, algae, bacteria, or fungi that influence the growth and development of biological and agricultural systems [22]. The key point of the definition is the putative chemicals produced by one plant and released into the environment to influence the growth and development of neighbor plants. If we can determine that the interference on target weeds is the result of chemicals exuded by rice, we can define any such interference as the allelopathic effect of the rice accession in question. In this paper, we quantified the intensity of competition between rice and barnyardgrass by target-neighbor mixed-culture. We demonstrate that by excluding uncontrolled soil factors this approach can separate the effects of allelopathy from resource competition.

## Results

### Competition intensity of the two rice accessions and BYG in rice/BYG mixed-cultures

The root length, plant height, and plant dry weight of BYG were significantly decreased in rice/BYG mixed-cultures relative to controls. The effect of PI on BYG growth was more intense than that of LE. Root length, plant height and plant dry weight of LE were significantly decreased in rice/BYG mixed-cultures relative to controls. However, the root length, plant height, and plant dry weight of PI were significantly increased in rice/BYG mixed-cultures relative to controls (Table 1).

**Table 1:** Morphological parameters of rice and barnyardgrass in rice/BYG mixed-cultures.

Plant	Culture mode	RL/cm	PH/cm	DW/g plant^−1^
BYG	CK (monoculture)	7.32±0.26	19.37±0.56	0.152±0.005
	Mixed with LE	5.01±0.15*	15.46±0.25*	0.115±0.003*
	Mixed with PI	2.66±0.17*	8.82±0.29*	0.0810±0.003*
PI	CK (monoculture)	8.73±0.18	36.24±0.56	0.412±0.005
	Mixed with BYG	10.26±0.09*	44.35±0.83*	0.472±0.003*
LE	CK (monoculture)	9.21±0.13	38.19±0.49	0.446±0.004
	Mixed with BYG	8.71±0.11*	35.22±0.51*	0.403±0.006*

RL – root length; PH – plant height; DW – plant dry weight; BYG – barnyardgrass; PI – rice accession PI312777; LE – rice accession Lemont.

* – significantly different from the control (*P*<0.05).

The RCI values of root length, plant height, and plant dry weight were negative for PI, indicating facilitation in PI/BYG mixed-cultures. However, the RCI values for LE were positive, indicating competition in LE/BYG mixed-cultures (Table 2). These results showed that these two rice accessions have different responses to BYG stress. The RCI values for BYG were positive, indicating competition in rice/BYG mixed-cultures. However, the RCI values for BYG in BYG/PI mixed-cultures were much higher than those in BYG/LE mixed-cultures, indicating that PI was more competitive against BYG than LE.

**Table 2:** Competition indices of rice and barnyardgrass in rice/BYG mixed-cultures.

Plant	RCI			CR		
	RL	PH	DW	RL	PH	DW
PI (mixed with BYG)	−0.1753	−0.2238	−0.1456	3.2342	2.6876	2.1498
LE (mixed with BYG)	0.05429	0.07777	0.09641	1.3818	1.1555	1.1943
BYG (mixed with PI)	0.6366	0.5447	0.4671	0.3092	0.3721	0.4652
BYG (mixed with LE)	0.3156	0.2019	0.2434	0.7237	0.8654	0.8373

RCI – Relative competition intensity; CR – Competitive Ratio; RL – root length; PH – plant height; DW – plant dry weight; BYG – barnyardgrass; PI – rice accession PI312777; LE – rice accession Lemont.

CR value indicates the ratio by which one plant is more competitive than another. The CR values of PI showed that one individual PI plant was as competitive as 3.2342 BYG plants with respect to root length, 2.6876 BYG plants with respect to plant height, and 2.1498 BYG plants with respect to plant dry weight (Table 2). The CR values showed that one LE individual was equal to about 1 BYG plant. The CR of PI was more than twice of Le.

**Figure 1:**
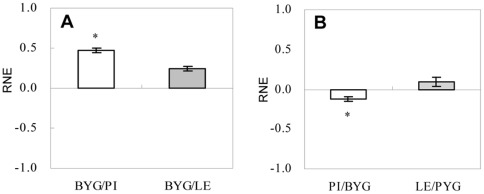
Relative neighbor effect (RNE) of each of the two rice accessions and barnyardgrass in rice/BYG mixed-cultures. A – RNE of BYG in mixed-cultures with PI and with LE. B – RNE of PI and LE in mixed-cultures with BYG. PI – rice accession PI312777; LE – rice accession Lemont. BYG – barnyardgrass. * –significantly different in different treatment groups (*P*<0.05).

The RNE values for BYG were positive, indicating that the plant-plant interactions between BYG and the two rice accessions involved competition in rice/BYG mixed-cultures (Fig. 1A). The RNE for BYG in PI/BYG mixed-cultures was significantly higher than that in LE/BYG mixed-cultures, indicating inter-specific competition of greater intensity between BYG and PI than between BYG and LE (Fig. 1A). In rice/BYG mixed-cultures, the RNE value was negative for PI but positive for LE, indicating that the plant-plant interactions involved facilitation for PI and competition for LE (Fig. 1B).

### Allelopathic effect and resource competition

The TB of PI on root length, plant height, and plant dry weight of BYG was about two times higher than that of LE in rice/BYG mixed-cultures (Table 3). However, the AE of PI on root length, plant height, and plant dry weight of BYG was about four times higher than that of LE.

**Table 3:** Inhibitory rate (%) of barnyardgrass in monoculture and rice/BYG mixed-cultures.

Index	TB		AE		AE/TB (%)	
	PI	LE	PI	LE	PI	LE
RL	57.79±0.24	30.05±0.11	40.88±0.15	8.36±0.14	70.74	27.82
PH	49.51±0.34	18.33±0.39	36.22±0.25	8.39±0.46	73.16	45.77
DW	43.42±0.002	21.71±0.004	38.98±0.001	11.02±0.003	89.77	50.76

Inhibitory rate (IR) was calculated as: IR = (1-treatment/control)×100%. TB – total biointerference, are the IRs of rice on BYG in rice/BYG mixed-cultures; AE – allelopathic effect, are the IRs of BYG monocultured in the residual solutions of rice/BYG mixed-cultures above. RL – root length; PH – plant height; DW – plant dry weight; BYG – barnyardgrass; PI – rice accession PI312777; LE – rice accession Lemont.

**Figure 2:**
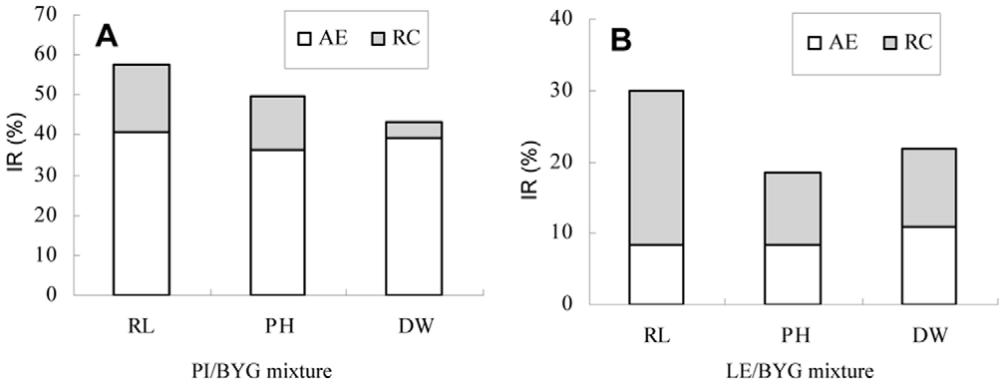
Separation of resource competition (RC) and allelopathic effect (AE) in rice/BYG mixed-cultures. A – Inhibitory rate (IR) of PI on BYG in PI/BYG mixture. B – Inhibitory rate (IR) of LE on BYG in LE/BYG mixture. TB – total biointerference. RC – resource competition. AE – allelopathic effect. TB (total bars) = RC (shaded bars)+AE (open bars). RL – root length; PH – plant height; DW – plant dry weight. PI – rice accession PI312777; LE – rice accession Lemont. BYG – barnyardgrass.

The residual solution from PI/BYG mixed-cultures had strong allelopathic effects on BYG growth. The AEs on root length, plant height, and plant dry weight accounted for 70.74%, 73.16%, and 89.77%, respectively, of the TB on BYG (Table 3). However, the AEs accounted for only 27.82%, 45.77%, and 50.76%, respectively, of the TB when BYG was cultured in the residual solutions from LE/BYG mixed-cultures. The allelopathic effect of PI was much higher than its resource competition in PI/BYG mixed-cultures, in which it was the predominant factor (Fig. 2A). The reverse was true for LE (Fig. 2B).

## Discussion

Significant differences in morphological indices (root length, plant height and dry weight) were observed between PI and LE growth in rice/BYG mixed-cultures relative to monocultured controls (Table 1). Analysis of competitive indices showed that PI was a stronger competitive potential against BYG than LE (Table 2). The plant-plant interaction was facilitation for PI but competition for LE (Fig. 1). Since rice and BYG are both Gramineae, they have morphological and phenological similarities and are believed to occupy similar niches. Causes other than resource competition must be responsible for the competitive differential between PI and LE. In order to determine the cause of the differential competition between the two rice accessions, the TB of each of the two rice accessions with respect to BYG were divided into two components, AE and RC. Results showed that the TB of PI on BYG was about twice that of LE, and the AE of PI on BYG was about four times that of LE (Table 3). AE was absolutely predominant in PI-BYG interactions (Fig. 2). The fact that PI has a more powerful interaction with BYG than LE suggests that it has strong allelopathic potential, which was reported by Dilday in his field tests [9], [10].

Because of methodological problems, conclusions made from many studies on allelopathy remain unconvincing. According to Inderjit and del Moral, it is physically impossible to separate allelopathy from resource competition in natural systems because any type of experimental design will create artificial conditions that do not occur in nature [12]. In a target-neighbor design that used corn-soybean mixed-cultures (with a finite amount of herbicide, atrazine, as a supposed allelochemical), soybeans showed increased growth at higher corn densities [18]. This was in contrast to the predicted effects of resource competition and was found to be due to uptake of atrazine by the corn plants, which decreased the amount available to the soybean targets. Experiments that used soil supplemented with gallic acid and hydroquinone as putative inhibitors and others that used soil taken from beneath and around black walnut trees, which are well known for phytotoxic effects on neighbor plants, both showed that phytotoxicity decreased as plant density increased, suggesting that the toxin was shared or diluted at high plant densities, giving each individual a proportionally lower dose [17]. These reductions in growth that occur at low but not high densities were in contrast to the then-prevailing hypothesis of resource competition. These studies provided convincing evidence for the presence of toxins (simulated allelochemicals) in soil. However, as the authors pointed out that in these study, allelopathic effect was distinguished rather than separated from resource competition [20]. One should not try to re-create impossible natural conditions to demonstrate the allelopathy phenomenon as it is almost impossible to do so. In our hydroponic experimental system we were able to separated allelopathy from resource competition by excluding the complexity involved in soil. Although the results of hydroponic experiments will differ from that of field experiments, it is obvious that the chemicals in the residual solution of PI/BYG mixed-cultures had a strong inhibitory effect on BYG growth.

A number of putative allelochemicals in rice have been reported such as long-chain fatty acid esters, benzaldehydes, terpenoids, momilactone, steroids, as well as phenolic acids [23]–[28]. However, it is commonly accepted that allelopathy is responsible for a complex of chemicals, rather than one specific solely as a result of interference [10], [27]–[32]. Kim et al. [33] reported that the inhibition of allelopathic rice (Kouketsumochi) on BYG was increased as BYG number increased in a mixed culture experiment, and suggested that Kouketsumochi had stronger allelopathic effects when grown under more competitive conditions. Hisashi [25] reported that the allelopathic activity of rice seedlings was significantly increased when rice and BYG were grown together than rice seedlings cultured independently. Under low nitrogen stress, inhibition of PI on BYG was enhanced and genes expression of PAL and P450 in PI were increased [34]. Fang et al. [35] reported that the expression of the genes associated with allelochemical synthesis and its detoxification were all up-regulated in PI when mixed cultured with BYG, indicated that BYG is not only a stressful factor but also a trigger in activating allelopathy in rice. These results confirmed that rice allelopathy is an inducible responsible mechanism that is associated with molecular regulation of secondary metabolic pathways.

The most significant result of our study is the discovery of an experimental design, target-neighbor mixed-culture in combination with competition indices, can successfully separate allelopathic effects from competition and quantify each contribution of allelopathy and competition in plant-plant interference. It is also an applicable approach in interpretation of intercropping system and/or crop-weed relationship in agricultural field. Since rice allelopathy is a quantitative inheritance [10], [36], [37] and is an inducible responsible mechanism, we should shift our attention discover ways to enhance the allelopathic potential of rice to combat weeds, which in turn will reduce the use of synthetic herbicides. More significant results have been documented showing that rice allelopathic potential could be induced and/or enhanced by exogenous salicylic acid, ferulic acid, *p*-coumaric acid, *p*-hydroxybenzonic acid, methyl jasmonate, methyl salicylate, as well as BYG exudates [25], [38]–[40]. Considering that the real-world use of a genetically modified allelopathic crop poses some environmental risk [41], we suggest that improvement of rice allelopathy by integrated regulation technology may be a practical and effective measure for integrated weed management, in the near future.

## Materials and Methods

The experiment was conducted in a greenhouse at the Agroecological Institute of Fujian Agriculture and Forestry University in Fuzhou, China. The temperature ranged from 25°C to 35°C, averaging 30°C during the trials. Allelopathic rice accession PI and its counterpart, non-allelopathic rice accession LE, were chosen as donor plants, and BYG (*Echinochloa crus-galli* L.), a Gramineae with morphological and phenological similarities to rice, was chosen as a receiver.

### Evaluation of competition intensity

The first experiment was designed to investigate the competition intensity of each of the two different allelopathic rice accessions and BYG using rice/BYG mixed-cultures in hydroponic solutions. The germinated seeds of the two rice accessions and BYG were sown in sand. Then uniform rice seedlings (3-leaf stage) and BYG seedlings (2-leaf stage) were transplanted into styrofoam plates with 40 perforated holes (5×8 holes of 5 cm×5 cm). The seedlings were stabilized with cotton plugs inserted into each hole. The styrofoam plates with seedlings were floated in a plastic basin (45×35×15 cm) containing 10-L Hoagland solution. Seven days after recovery, 20 rice seedlings and 20 BYG seedlings were chosen for mixed-culture in alternating rows (8 rows of 5 plants each). New 10-L Hoagland solution was supplied and pH was adjusted to 5.5. The controls were 20-seedling monocultures containing either of the two rice accessions or BYG. The treatments were performed in triplicate in completely randomized design. Additional distilled water was added daily to each pot to maintain the 10 L volume of the culture solution. Seven days after treatment, all plants were harvested, and root length and plant height were measured. Then the plants were oven dried at 120°C for 30 min and at 80°C for 48 h. Plant dry weights were recorded.

### Separation of allelopathic effect and competition

The second experiment was designed to separate allelopathic effects (AE) from the total biointerference (TB). The culture mode was the same as in the first experiment. During the first step, 20 rice seedlings (3-leaf stage) and 10 BYG seedlings (2-leaf stage) were mixed-cultures in alternate rows in 10-L Hoagland solution. The controls were monocultures of 10 BYG seedlings. Seven days after treatment, root length, plant height, and plant dry weight of BYG seedlings were obtained as in the first experiment. Results were defined as TB of each of the two rice accession on the associated BYG. During the second step, the culture solutions (containing root exudates of both rice accessions in rice/BYG mixed-cultures, the putative allelochemicals) of each of the basins described above were collected. The levels of nitrogen (N), phosphate (P), and potassium (K) in these solutions were measured. The N, P, K were adjusted to normal level of 10-L Hoagland solution using NH_4_NO_3_, KH_2_PO_4_, and K_2_SO_4_ and pH was adjusted to 5.5. Ten BYG seedlings (2-leaf stage) were transplanted into these solutions. The controls were 10 BYG seedlings in 10-L Hoagland solution. The results of this step were defined as AE of each of the two rice accession on the associated BYG because any actual competition between rice and BYG had been removed. The difference between this treatment and the control could only have come from the allelochemicals in the residual solutions. The treatments were performed in triplicate in completely randomized design. Additional distilled water was added daily to each pot to maintain the 10-L volume of the culture solution. Seven days after treatment, the root lengths, plant heights, plant dry weights of BYG seedlings were obtained as in the first experiment.

### Data analysis

In the first experiment, the root length, plant height, and plant dry weight of the two rice accessions and BYG were used as indices of plant competition as follows.

Relative competition intensity (RCI) was used to evaluate the competition between the two rice accessions and BYG, respectively and was calculated as follows [42]:
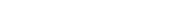



Here, P_mono_ represents the performance indices (root length, plant height and plant dry weight) of a plant in monoculture (controls of two rice accessions and BYG, respectively) and P_mix_ represents the performance indices of a plant in a mixed-cultures (treatments). Positive RCI values indicate competitive inhibition and the negative values indicate competitive facilitation.

The competitive ratio (CR) was used to compare the competitive abilities of rice and BYG. It was calculated as follows [43]:
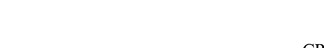



Here, CR_rb_ is the competitive ratio of rice on BYG and CR_br_ is the competitive ratio of BYG on rice. By definition, CR_rb_×CR_br_ = 1, so CR values indicate the ratio by which one plant is more competitive than the other.

The relative neighbor effect (RNE) was used to indicate the inter-specific competitive effect on each of the two rice accessions and BYG for plant dry weight. It was calculated as follows [44]:
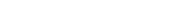



Here, P_max_ is the highest value of (P_mono_, P_mix_). RNE is a modified version of RCI because RCI is not symmetrical around zero. RNE ranges from −1 to +1, with negative values indicating facilitation and positive values indicating competition.

In the second experiment, the inhibitory rate (IR) was used to assess the inhibition of each of the two rice accessions on the growth of BYG. The IR was calculated as follows:
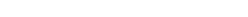



IR>0 and IR<0 indicate inhibitory effects and stimulatory effects, respectively. The IRs from the mixed-cultures represent the TB of each of the two rice accessions on BYG and IRs from the monoculture represent the AE of each of the two rice accessions on BYG. Therefore, resource competition (RC) = TB−AE.

All experimental data are presented as mean ± standard error (SE). They were subjected to a one-way analysis of variance (ANOVA) followed by the least significant difference (LSD) at a 5% level of probability. The statistical analysis was performed using the DPS data processing system [45].
